# Cucurbitaceae COld Peeling Extracts (CCOPEs) Protect Plants From Root-Knot Nematode Infections Through Induced Resistance and Nematicidal Effects

**DOI:** 10.3389/fpls.2021.785699

**Published:** 2022-01-26

**Authors:** Jonas De Kesel, Eva Degroote, Radisras Nkurunziza, Richard Raj Singh, Kristof Demeestere, Karen De Kock, Riska Anggraini, Jasper Matthys, Eva Wambacq, Geert Haesaert, Jane Debode, Tina Kyndt

**Affiliations:** ^1^Department of Biotechnology, Faculty of Bioscience Engineering, Ghent University, Ghent, Belgium; ^2^Department of Green Chemistry and Technology, Faculty of Bioscience Engineering, Ghent University, Ghent, Belgium; ^3^Department of Plants and Crops, Faculty of Bioscience Engineering, Ghent University, Ghent, Belgium; ^4^Flanders Research Institute for Agriculture, Fisheries and Food (ILVO), Merelbeke, Belgium

**Keywords:** induced resistance, rice, tomato, *Meloidogyne* spp., plant protection products, valorization of waste streams

## Abstract

With nematicides progressively being banned due to their environmental impact, an urgent need for novel and sustainable control strategies has arisen. Stimulation of plant immunity, a phenomenon referred to as “induced resistance” (IR), is a promising option. In this study, Cucurbitaceae COld Peeling Extracts (CCOPEs) were shown to protect rice (*Oryza sativa*) and tomato (*Solanum lycopersicum*) against the root-knot nematodes *Meloidogyne graminicola* and *Meloidogyne incognita*, respectively. Focusing on CCOPE derived from peels of melon (*Cucumis melo* var. *cantalupensis*; mCOPE), we unveiled that this extract combines an IR-triggering capacity with direct nematicidal effects. Under lab conditions, the observed resistance was comparable to the protection obtained by commercially available IR stimuli or nematicides. Via mRNA sequencing and confirmatory biochemical assays, it was proven that mCOPE-IR in rice is associated with systemic effects on ethylene accumulation, reactive oxygen species (ROS) metabolism and cell wall-related modifications. While no negative trade-offs were detected with respect to plant growth or plant susceptibility to necrotrophic pests or pathogens, additional infection experiments indicated that mCOPE may have a predominant activity toward biotrophs. In summary, the presented data illustrate a propitious potential for these extracts, which can be derived from agro-industrial waste streams.

## Introduction

Causing annual crop yield losses worth an estimated 100 billion US dollar, plant-parasitic nematodes are a widespread agricultural problem (Bernard et al., 2017). The group of sedentary endoparasitic nematodes is considered as most damaging for agriculture (Jones et al., 2013), with cyst nematodes and root-knot nematodes (*Meloidogyne* spp.) being its main representatives (Lambert and Bekal, 2002). The latter group of nematodes has a worldwide prevalence and is thought to infect nearly all plant species. Upon infection of host roots, local swellings – referred to as “galls” or “root-knots” – are induced by *Meloidogyne* spp. In these macroscopic structures, nematodes multiply and extract nutrients from the plant’s protoxylem and protophloem. As a result, *Meloidogyne* spp. can drastically affect growth, development and yield of the parasitized plant (Karssen et al., 2013; Mantelin et al., 2017; Rusinque et al., 2021). Correspondingly, root-knot nematodes are known to be highly damaging for both rice and tomato cultivation, being the fourth and tenth most cultivated crops worldwide, respectively (Food and Agriculture Organization Corporate Statistical Database [FAOSTAT], 2021). Depending on the cultivar, applied agricultural practices and (a)biotic circumstances, *Meloidogyne graminicola* (*Mg*) has been attributed to rice yield losses varying between 5 and 73% (Matias and Prot, 1995; Tandingan et al., 1996; Win et al., 2015; Kumar et al., 2020). Similarly, infections with *Meloidogyne incognita* (*Mi*) pose a major threat to tomato farming, with reported yield losses of up to 80% (Seid et al., 2015). Moreover, the importance of nematode species such as *Mg* is expected to considerably increase in the coming years. That is because rice cultivation via inundated “paddy fields” is envisioned to be replaced by the “aerobic rice cultivation strategy”, as the former has significant ecological costs concerning freshwater usage and methane emissions (Solomon et al., 2007; Consultative Group for International Agricultural Research [CGIAR], 2011; Templeton and Bayot, 2011; Priyanka et al., 2012). However, aerobic rice production still results in lower yields, a phenomenon that has been attributed to increased prevalence and infectivity of *Mg* in aerobic soils (De Waele and Elsen, 2007; Kreye et al., 2009; Priyanka et al., 2012). To control nematodes such as *Meloidogyne* spp., the use of chemical nematicides (being pesticides that target nematodes) is possible, though unfavorable from an ecological point-of-view. Indeed, having a high and broad-spectrum toxicity, intense usage of these pesticides is often associated with severe environmental effects. As a result, various commercial nematicides have been banned in recent years (Oka, 2020; Desaeger et al., 2021).

A more environmentally friendly strategy for nematode control is “induced resistance” (IR) (Oka et al., 2000; Dias-Arieira et al., 2013; Mantelin et al., 2017). This is a plant phenotype that is characterized by a conditioned state of enhanced defensive capacity against upcoming pathogens and pests, established upon contact with so-called “IR stimuli” (Mauch-Mani et al., 2017; De Kesel et al., 2021). IR can result in durable and long-lasting resistance, while not affecting non-pathogenic species (Alexandersson et al., 2016). For both the rice-*Mg* and tomato-*Mi* pathosystem, various IR stimuli have been shown to effectively lower nematode parasitism, albeit nearly uniquely under lab conditions only (Dababat and Sikora, 2007; Vos et al., 2013; Ji et al., 2015; Huang et al., 2016; Martínez-Medina et al., 2017; Pocurull et al., 2020; Singh et al., 2020b; Desmedt et al., 2021; Soares and Dias-Arieira, 2021).

Another alternative to accomplish nematode control with limited environmental impact is the development of nematicides with minimal or no ecotoxicity (Oka, 2020). Various extracts of Cucurbitaceae plant or fruit parts, for instance, have been shown to possess nematicidal effects to *Mi* (Elbadri et al., 2008; Gad et al., 2018; Alam and El-Nuby, 2019). Interestingly, plant-derived solutions are rarely associated with ecological toxicity due to their organic nature and corresponding biodegradability (Šernaitė, 2017). Furthermore, plant extracts are generally complex mixtures of various bioactive ingredients, limiting the risk of pathogens acquiring resistance (Šernaitė, 2017). Indeed, simultaneous acquisition of effective resistance against multiple active ingredients by a pest or pathogen is considerably less likely than overcoming a single-ingredient pesticide (Šernaitė, 2017). Nemguard^®^ is an example of a successfully commercialized extract of garlic (*Allium sativum*) that is used to control *Meloidogyne* spp. specifically (Ladurner et al., 2014; Eder et al., 2021). Interestingly, combined IR-triggering and direct anti-pathogenic effects have also been demonstrated for some (plant-derived) compounds (Kagale et al., 2004; Godard et al., 2009; Hassan et al., 2009; Sangeetha et al., 2013; Krzyzaniak et al., 2018). Birch et al. (1993) illustrated that the rare sugar 2,5-dihydroxymethyl-3,4-dihydroxypyrrolidine (DMDP) inhibited hatching of *Globodera pallida* and immobilized *Globodera rostochiensis* juveniles, while DMDP-IR in tomato was shown to result in a lower host susceptibility to *Meloidogyne* spp. To our knowledge, DMDP is the only compound known to result in nematode control via a combination of IR elicitation and direct anti-nematode effects.

In the presented study, it was investigated whether Cucurbitaceae COld Peeling Extracts (CCOPEs) can be used to control infection of rice and tomato with *Mg* and *Mi*, respectively. Extracts of various cucurbits were found to be potent IR stimuli, while CCOPE derived from melon (*Cucumis melo* var. *cantalupensis*; mCOPE) was demonstrated to be a strong nematicidal agent as well. Via mRNA sequencing and independent biochemical validation assays, the mode-of-action of mCOPE-IR in rice was unveiled. Moreover, mCOPE’s effectiveness was compared to some commercially available IR stimuli or nematicides. Finally, we investigated whether the melon extract could also protect other plants against various pests and pathogens.

## Results

### Cucurbitaceae COld Peeling Extracts (CCOPEs) Induce Resistance Against Root-Knot Nematodes in Rice and Tomato

**FIGURE 1:**
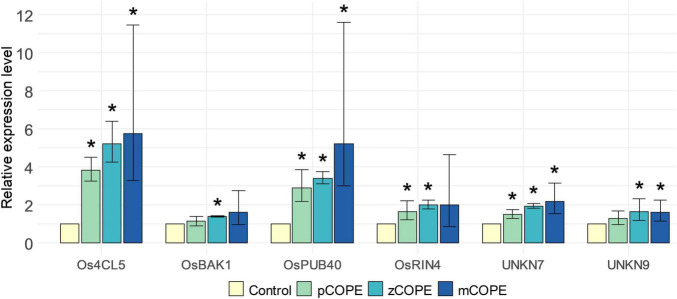
Cucurbitaceae COld Peeling Extracts (CCOPEs) are promising candidates to trigger induced resistance (IR). Rice cell suspension cultures (RCSCs) were incubated for 4 h with the buffer used for CCOPE preparation (Control) or CCOPE derived from pumpkin (*Cucurbita moschata* cv. Musquee de Provence; pCOPE), zucchini (*C. pepo* var. *cylindrica*; zCOPE) or melon (*Cucumis melo* var. *cantalupensis*; mCOPE). Expression levels of the six screening genes identified by De Kesel et al. (2020) were determined by RT-qPCR and are expressed relative to the mock-treated cells. Error bars indicate the 95% confidence interval. Asterisks indicate statistically significant differences upon comparison of CCOPE- and mock-treated cells. Statistical results and 95% confidence intervals were obtained via the Rest2009 software (Pfaffl, 2001) (*p* < 0.05).

The screening platform developed by De Kesel et al. (2020) was used to investigate potential IR-establishing effects of various CCOPEs. Rice cell suspension cultures (RCSCs) were treated with candidate solutions to evaluate the induction of a validated pattern-triggered immunity (PTI)-marker gene set. This *in vitro* transcriptional stimulation has been shown to be a useful proxy to unveil an IR-inducing capacity of assayed products (De Kesel et al., 2020). Extracts derived from pumpkin (*Cucurbita moschata* cv. Musquee de Provence; pCOPE), zucchini (*C. pepo* var. *cylindrica*; zCOPE) or melon (*Cucumis melo* var. *cantalupensis*; mCOPE) peelings were all found to be promising IR stimuli. Indeed, convening with the threshold set by De Kesel et al. (2020), at least four of the six screening genes were significantly upregulated in the RCSCs upon treatment with the evaluated CCOPEs (Figure 1).

**FIGURE 2:**
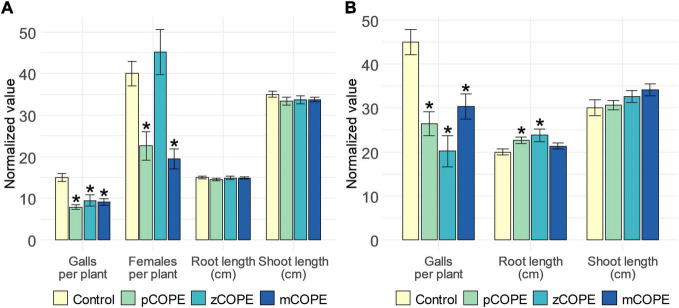
Foliar application of Cucurbitaceae COld Peeling Extracts (CCOPEs) enhances the resistance of rice and tomato against the root-knot nematodes *Meloidogyne graminicola* (*Mg*) and *Meloidogyne incognita* (*Mi*), respectively. **(A)** Normalized infection and growth parameters, as assessed for rice plants 14 days post inoculation with 250 *Mg* second-stage juveniles (J2s). **(B)** Normalized infection and growth parameters, as assessed for tomato plants 28 days post inoculation with 250 *Mi* J2s. **(A,B)** One day before inoculation, shoots of 14-days-old plants were treated with the buffer used for CCOPE preparation (Control) or CCOPE derived from pumpkin (*Cucurbita moschata* cv. Musquee de Provence; pCOPE), zucchini (*C. pepo* var. *cylindrica*; zCOPE) or melon (*Cucumis melo* var. *cantalupensis*; mCOPE). Error bars represent the standard error of the mean. Asterisks indicate statistically significant differences upon comparison of CCOPE- and mock-treated cells. Statistical differences were determined via a two-sided heteroscedastic *t*-test (*p* < 0.05).

Via rice-*M. graminicola* (*Mg*) and tomato–*M. incognita* (*Mi*) infection studies, it was examined whether CCOPEs could result in effective systemic protection *in planta*. In line with the *in vitro* results (Figure 1), foliar CCOPE treatment 1 day before nematode inoculation significantly enhanced the root resistance of rice and tomato against the root-knot nematodes under study (Figure 2). In both hosts, all three evaluated CCOPEs resulted in a significantly lower number of galls per root system when compared to mock-treated control plants. Additionally, the number of females were counted for the infected rice plants: both in pCOPE- and mCOPE-treated plants, this infection parameter was found to be significantly reduced (*p* = 1.89 × 10^–3^ and *p* = 3.53 × 10^–4^, respectively). Interestingly, tomato root lengths increased upon pCOPE or zCOPE treatment (Figure 2B). Rice root or shoot lengths, on the other hand, were not consistently affected upon CCOPE treatment (Figure 2A). However, upon investigation of individual repetitions and/or data obtained in other experiments, treatment with pCOPE or zCOPE was found to be sporadically associated with impaired growth of rice plants (Supplementary Figure 1).

Additional rice-*Mg* infection assays were done to evaluate the IR-inducing capacity of other CCOPEs. Although a significantly lowered susceptibility could also be observed upon treatment with CCOPEs extracted from peels of butternut pumpkin (*C. moschata* cv. Butternut) or cucumber (*Cucumis sativus*), moderate to severe phytotoxicity was induced by these extracts (Supplementary Figure 2). As mCOPE consistently led to significant protection in both crops, and never had a negative effect on plant growth or development, this extract was selected for a more profound investigation.

### mCOPE Acts as a Nematicidal Agent on Root-Knot Nematodes

**FIGURE 3:**
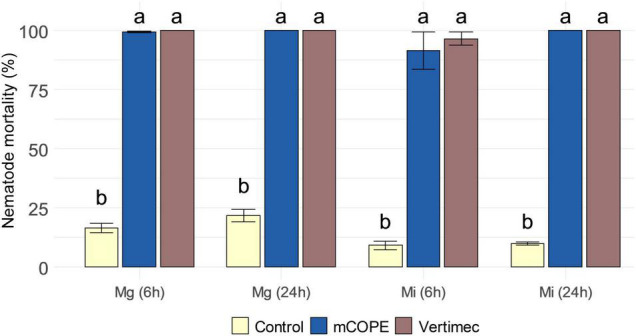
Cucurbitaceae COld Peeling Extract (CCOPE) derived from melon (*Cucumis melo* var. *cantalupensis*; mCOPE) is strongly nematicidal. Second-stage juveniles (J2s) of *Meloidogyne graminicola* (*Mg*) and *Meloidogyne incognita* (*Mi*) were incubated in the buffer used for CCOPE preparation (Control), mCOPE or 0.2% (v/v) Vertimec. The latter is a commercial formulation of the nematicidal compound abamectin (Pitterna et al., 2009; Li et al., 2018) and was used as positive control. After 6 and 24 h of incubation, nematode viability was assessed. Error bars represent the standard error of the mean. Letters indicate significant differences upon investigation of all possible pairwise comparisons. Statistical differences were determined via a two-sided heteroscedastic *t*-test (*p* < 0.05).

During the above-discussed infection experiments (Figure 2), plant roots were inoculated 1 day post foliar treatment. As such, potential contact between the applied extracts and the inoculated nematodes could be limited. Nevertheless, various other cucurbit extracts are known to possess nematicidal properties to *Mi* (Elbadri et al., 2008; Gad et al., 2018; Alam and El-Nuby, 2019). To evaluate the nematicidal activity of mCOPE, second-stage juveniles (J2s) of *Mg* and *Mi* were incubated for 6 and 24 h in this extract. Figure 3 illustrates that mCOPE exhibits obvious nematicidal properties. Indeed, near-complete mortality rates were observed upon mCOPE incubation, similarly as observed for the positive control Vertimec, a commercial formulation of the nematicidal compound abamectin (Pitterna et al., 2009; Li et al., 2018).

### mCOPE-IR in Rice Is Associated With Activation of Plant Defense Pathways and Alterations in Ethylene, Reactive Oxygen Species, and Cell Wall Metabolism

Considering the major importance of rice as staple crop over tomato and its well-annotated genome, the mode-of-action of mCOPE-IR was further investigated in this monocot species via mRNA sequencing. Root samples were investigated 1 and 4 days post foliar treatment (1 and 4 dpt, respectively), allowing an assessment of systemic effects. 1,306 and 788 genes were found to be induced or repressed at 1 dpt, respectively, whereas these numbers equaled 2,341 and 1,224 for the 4 dpt analysis. 741 and 321 genes were found to be consistently induced or repressed at both time points. Multiple pathways contributing to plant biotic defense responses were found to be induced (Table 1), confirming mCOPE’s IR-triggering capacity. Amongst others, induction of phytohormone signaling through ethylene (ET) and jasmonic acid (JA), as well as cell wall biosynthesis and reactive oxygen species (ROS) metabolism, were found to be induced in roots of mCOPE-treated rice plants (Table 1). Moreover, MapMan and gene ontology (GO)-enrichment analyses revealed that genes involved in “plant–pathogen interactions,” “response to stress,” and “biosynthesis of secondary metabolites” were significantly enriched among the mCOPE-induced gene set (Table 1). Per-gene transcription levels and complete outcomes of MapMan and GO-enrichment analyses can be found in Supplementary Tables 1, 2, respectively.

**TABLE 1:** Foliar treatment with Cucurbitaceae COld Peeling Extract (CCOPE) derived from melon (*Cucumis melo* var. *cantalupensis*; mCOPE) leads to systemic defense induction in rice.

**Terms upregulated at 1 dpt**		
**MapMan**		
**Bin ID**	**Bin name**	***p*-value**

10	Cell wall	<1 × 10^–20^
26.12	Peroxidases	1.43 × 10^–5^
20.1	Biotic stress	3.13 × 10^–4^
27.3.32	WRKY transcription factors	5.07 × 10^–3^
17.3.32	Hormone metabolism ethylene ACC synthase	2.33 × 10^–2^
17.7	Hormone metabolism jasmonate	3.10 × 10^–2^
21.2	Glutathione-ascorbate redox system	4.24 × 10^–2^

**Gene ontology**		

**Term ID**	**Term name**	***p*-value**

GO:0071554	Cell wall organization or biogenesis	1.05 × 10^–6^
GO:0009698	Phenylpropanoid metabolic process	1.30 × 10^–2^

**KEGG pathway**		

**Term ID**	**Term name**	***p*-value**
KEGG:04626	Plant–pathogen interaction	1.84 × 10^–4^
KEGG:00940	Phenylpropanoid biosynthesis	3.52 × 10^–4^
KEGG:01110	Biosynthesis of secondary metabolites	2.80 × 10^–2^

**Terms upregulated at 4 dpt**		

**MapMan**		

**Bin ID**	**Bin name**	***p*-value**

26.12	Peroxidases	2.42 × 10^–9^
27.3.32	WRKY transcription factors	7.38 × 10^–5^
16.2	Secondary metabolism phenylpropanoids	8.28 × 10^–5^
10.7	Cell wall modification	2.84 × 10^–3^
17.5.1.1	Hormone metabolism ethylene ACC synthase	5.89 × 10^–3^
20.1	Biotic stress	8.17 × 10^–3^

**Gene ontology**		

**Term ID**	**Term name**	***p*-value**

GO:0042744	Hydrogen peroxide catabolic process	2.93 × 10^–12^
GO:0071669	Plant-type cell wall organization or biogenesis	1.74 × 10^–4^
GO:0006950	Response to stress	2.94 × 10^–4^
GO:0031347	Regulation of defense response	4.27 × 10^–2^

**KEGG pathway**		

**Term ID**	**Term name**	***p*-value**

KEGG:00940	Phenylpropanoid biosynthesis	1.51 × 10^–9^
KEGG:04626	Plant–pathogen interaction	9.73 × 10^–7^
KEGG:01110	Biosynthesis of secondary metabolites	3.18 × 10^–2^

*The rice root transcriptome was investigated 1 and 4 days post mCOPE treatment (1 and 4 dpt, respectively) and compared with buffer-treated control plants. Upon treatment, all plants were 14 days old. This table shows a selection of defense-associated terms obtained via MapMan (Thimm et al., 2004) and gene ontology (GO)-enrichment (Reimand et al., 2016) analyses on significantly upregulated genes after comparing gene transcription levels of mCOPE- vs. mock-treated plants. ACC, 1-aminocyclopropane-1-carboxylic acid, an ethylene precursor. Per-gene transcription levels and complete outcomes of MapMan and GO-enrichment analyses can be found in Supplementary Tables 1, 2, respectively.*

**FIGURE 4:**
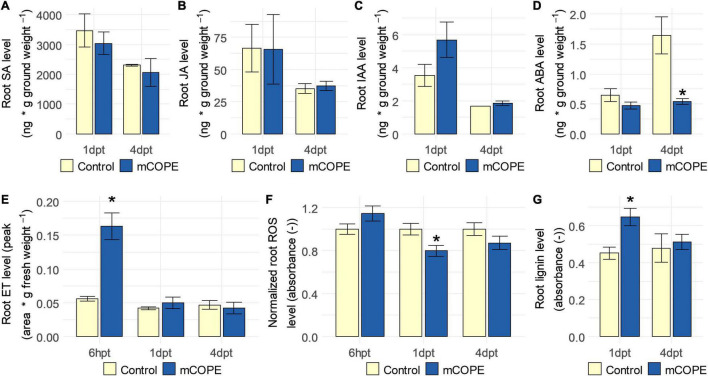
Establishment of induced resistance (IR) via Cucurbitaceae COld Peeling Extract (CCOPE) derived from melon (*Cucumis melo* var. *cantalupensis*; mCOPE) is associated with ethylene (ET) accumulation, lowered abscisic acid (ABA) concentrations, decreased levels of reactive oxygen species (ROS) and a transient increase in lignin content in roots of rice plants. Shoots of 14-days-old rice plants were treated with the buffer used for CCOPE preparation (Control) or mCOPE. Six hours, 1 day and/or 4 days later (6 hpt, 1 dpt, and 4 dpt, respectively), roots were collected to quantify levels of **(A)** salicylic acid (SA), **(B)** jasmonic acid (JA), **(C)** indole-3-acetic acid (IAA), **(D)** ABA, **(E)** ET, **(F)** ROS, and/or **(G)** lignin. **(A–G)** Error bars represent the standard error of the mean. Asterisks indicate significant differences upon comparison of mCOPE-treated plants with same-aged, mock-treated control plants. Statistical differences were determined via a two-sided heteroscedastic *t*-test (*p* < 0.05). Asterisks indicate statistically significant differences upon comparison of CCOPE- and mock-treated cells.

To independently validate the transcriptomic findings (Table 1), phytohormone, ROS and lignin levels were quantified in rice roots upon foliar mCOPE treatment. In correspondence with the mRNA sequencing experiment, this was done at 1 and 4 dpt. However, as systemic accumulation of ET (Singh et al., 2020b) and ROS (Huang et al., 2015) can occur relatively quickly upon IR stimulus treatment, the two cited metabolites were quantified additionally at 6 h post treatment (6 hpt). In line with the mRNA sequencing results (Table 1), ET levels were increased in roots of mCOPE treated plants at 6 hpt (*p* = 1.41 × 10^–3^) (Figure 4). No other changes in phytohormone levels were detected, except for a significant reduction in abscisic acid (ABA) levels at 4 dpt (*p* = 2.66 × 10^–2^) (Figure 4). Moreover, significantly lower levels of ROS were detected at 1 dpt (*p* = 9.66 × 10^–3^; Figure 4F), while lignin quantification revealed a transient increase of this biomolecule at 1 dpt (*p* = 6.97 × 10^–4^; Figure 4G). The latter two biochemical assessments were also in line with the transcriptomic data presented in Table 1.

**FIGURE 5:**
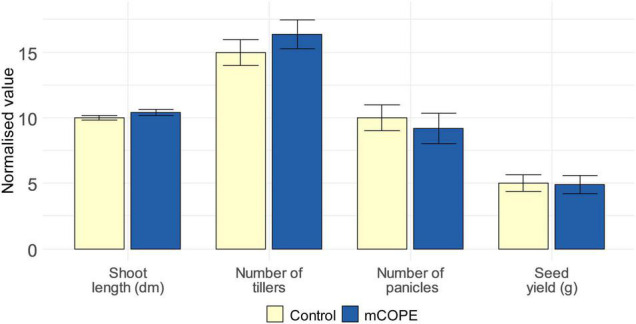
Repetitive treatments with Cucurbitaceae COld Peeling Extract (CCOPE) derived from melon (*Cucumis melo* var. *cantalupensis*; mCOPE) do not affect rice growth or development. Rice plants were grown under greenhouse conditions and foliarly treated on a biweekly basis with the buffer used for CCOPE preparation (Control) or mCOPE. The illustrated data were assessed when seeds were ready to be harvested (i.e., after a growth period of approximately 4 months). Error bars represent the standard error of the mean. No significant differences were detected upon comparison of mCOPE-treated plants with mock-treated control plants using a two-sided heteroscedastic *t*-test (*p* < 0.05).

As stated by Martínez-Medina et al. (2016), relevant ecological assessments are needed to evaluate fitness-related costs and benefits associated with IR establishment. This may be of particular interest when the IR phenotype under study is characterized by intense defense induction upon contact with the IR stimulus, while not (yet) being challenged by a pest or pathogen (De Kesel et al., 2021). Such direct effects were revealed by our transcriptome analysis (Table 1) and the subsequent biochemical validations (Figure 4). Therefore, growth and yield of rice plants was investigated upon lifelong biweekly mCOPE treatment. Despite the intense defense elicitation as unveiled by mRNA sequencing, no negative long-term effects were observed for repetitively treated plants (Figure 5). Indeed, plant height, number of tillers, number of panicles and seed yield were all unaffected upon comparison with mock-treated control plants. These findings concur with the growth data presented in Figure 2 and Supplementary Figure 1.

### mCOPE Functions as Agronomically Relevant Nematode Control Agent

**FIGURE 6:**
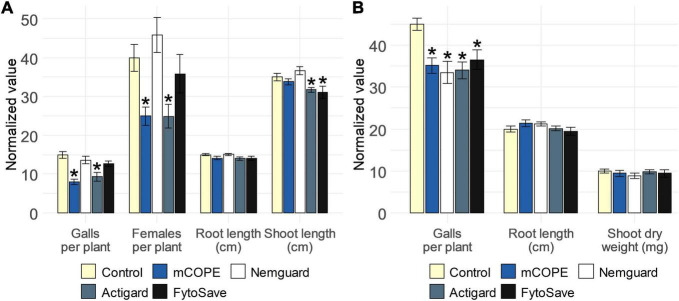
Cucurbitaceae COld Peeling Extract (CCOPE) derived from melon (*Cucumis melo* var. *cantalupensis*; mCOPE) leads to similar or better protection for the pathosystems **(A)** rice-*Meloidogyne graminicola* (*Mg*) and **(B)** tomato-*Meloidogyne incognita* (*Mi*) when compared to commercially available nematicides or IR stimuli. **(A)** Normalized infection and growth parameters, as assessed for rice plants 14 days post inoculation with 250 *Mg* second-stage juveniles (J2s). **(B)** Normalized infection and growth parameters, as assessed for tomato plants 28 days post inoculation with 250 *Mi* J2s. **(A,B)** One day before inoculation, shoots of 14-days-old plants were treated with the buffer used for CCOPE preparation, mCOPE, Actigard^®^ [250 μM benzothiadiazole (BTH)] or FytoSave^®^ [0.5% (v/v), corresponding with a chito-oligosaccharides and oligogalacturonides (COS-OGA) concentration of 12.5 g/L]. A solution of 0.133 g/L Nemguard^®^ was administered via soil drenching 1 day before nematode inoculation according to the manufacturer’s instructions. Error bars represent the standard error of the mean. Asterisks indicate significant differences upon comparison of all treatments with same-aged, mock-treated control plants. Statistical differences were determined via a two-sided heteroscedastic *t*-test (*p* < 0.05). Asterisks indicate statistically significant differences upon comparison of CCOPE- and mock-treated cells.

To find out whether mCOPE-triggered protection against root-knot nematodes could be agronomically relevant for rice and tomato cultivation, a comparison with commercially available nematicides or IR stimuli was performed. The garlic extract Nemguard^®^, marketed for the control of root-knot nematodes specifically (Ladurner et al., 2014; Eder et al., 2021), was used as reference nematicide. The IR stimuli included in this study were Actigard^®^, a commercial formulation of benzothiadiazole (BTH; Schurter et al., 1990) and FytoSave^®^, a preparation of chito-oligosaccharides and oligogalacturonides (COS-OGA) complexes (van Aubel et al., 2014; Clinckemaillie et al., 2017). Both IR stimuli and/or their active ingredients had previously been shown to result in effective protection of rice against *Mg* (Nahar et al., 2011; Singh et al., 2019; Soares and Dias-Arieira, 2021). Furthermore, BTH-IR had also been shown to protect tomato against *Mi* (Melillo et al., 2014). The effect of COS-OGA-IR against *Mi*, on the other hand, had not yet been studied for tomato. However, van Aubel et al. (2016) reported that this crop is prone to IR-elicitation via COS-OGA, as reduced susceptibility to *Leveillula taurica* was observed under greenhouse conditions. Our results indicated that, under lab conditions, mCOPE worked as good, or even better, than the commercially available products (Figure 6). Indeed, with respect to rice, Actigard^®^-IR was effective in protecting rice from *Mg*, while Nemguard^®^ did not affect the infectivity of this nematode. FytoSave^®^ only led to a near-significant reduction in the number of galls for this pathosystem (*p* = 5.65 × 10^–2^). Moreover, both Actigard^®^ and FytoSave^®^ resulted in reduced rice shoot length, while mCOPE did not (Figure 6A). Concerning tomato, all tested compounds led to a significant reduction in nematode infection levels, while not affecting plant growth (Figure 6B).

### mCOPE May Be Predominantly Active Against Biotrophs

**FIGURE 7:**
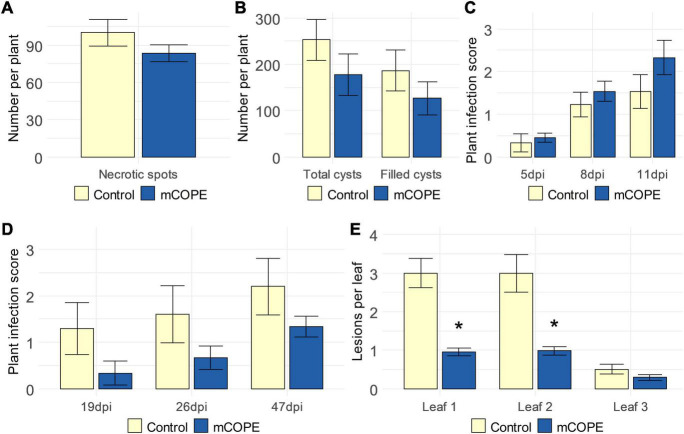
Cucurbitaceae COld Peeling Extract (CCOPE) derived from melon (*Cucumis melo* var. *cantalupensis*; mCOPE) may predominantly lead to effective protection against biotrophs. **(A)** Number of necrotic spots observed in rice roots, 25 days post inoculation with 250 *Pratylenchus zeae* second-stage juveniles (J2s). **(B)** Total number of cysts and egg-filled cysts found in the soil surrounding sugar beets (*Beta vulgaris* cv. Amarok), 6 weeks post inoculation with 300 *Heterodera schachtii* J2s. **(C)** Infection scores as assessed 5, 8, and 11 days post inoculation of strawberry (*Fragaria* × *ananassa*) with *Botrytis cinerea* (5, 8, and 11 dpi, respectively). **(D)** Infection scores as assessed nineteen, 26 and 47 days post inoculation of fodder beet (*B. vulgaris* cv. Brunium) with *Rhizoctonia solani* (19, 26, and 47 dpi, respectively). **(E)** Number of lesions in the first, second, and third leaf of tomato plants, 4 days post inoculation with *Pseudomonas syringae*. **(A–C,E)** One day before inoculation, plant shoots were treated with mCOPE. **(D)** mCOPE treatment was done at the moment of sugar beet transfer into *R. solani*-inoculated sand. **(A–E)** Error bars represent the standard error of the mean. Asterisks indicate significant differences upon comparison of mCOPE-treated plants with same-aged control plants that were treated with the buffer. Statistical differences were determined via a two-sided heteroscedastic *t*-test (*p* < 0.05). Asterisks indicate statistically significant differences upon comparison of CCOPE- and mock-treated cells.

To investigate the activity spectrum of mCOPE-IR, additional infection experiments were conducted using various pathosystems that included pests or pathogens with different life styles. The migratory nematode *Pratylenchus zeae* and the cyst nematode *Heterodera schachtii* were used to challenge rice and sugar beet (*Beta vulgaris* cv. Amarok), respectively. The former parasite induces cell death and necrotic lesions due to its feeding strategy (Moens and Perry, 2009), while the latter is characterized by a biotrophic life style (Habash et al., 2017). Resistance to *Botrytis cinerea* and *Rhizoctonia solani* – both being necrotrophic fungi (Williamson et al., 2007; Foley et al., 2016) – was investigated upon mCOPE treatment of strawberry (*Fragaria* × *ananassa*) and fodder beet (*B. vulgaris* cv. Brunium), respectively. Finally, tomato was challenged by *Pseudomonas syringae*, a bacterial pathogen with a predominantly biotrophic life style (Kraepiel and Barny, 2016). Effective mCOPE-IR could not be established against any of the studied necrotrophs (Figures 7A,C,D). On the other hand, next to lowering the infectivity of the biotrophs *Mg* and *Mi* (Abad et al., 2008; Htay et al., 2016) upon infection of rice and tomato, respectively (Figure 2), mCOPE pretreatment also led to protection in tomato when challenged by the biotrophic pathogen *P. syringae* (Figure 7E). As a result, *H. schachtii* was the only investigated parasite with a biotrophic life style whose infection levels were not affected upon mCOPE-IR establishment in the challenged host (Figure 7B). Although more research is needed, these data seem to indicate that mCOPE may have a predominant activity against biotrophs.

## Discussion

In this research, we have demonstrated that various Cucurbitaceae COld Peeling Extracts (CCOPEs) can be used to protect rice and tomato from infections with the root-knot nematodes *M. graminicola* (*Mg*) and *M. incognita* (*Mi*), respectively (Figure 2). Furthermore, CCOPE derived from melon (*Cucumis melo* var. *cantalupensis*; mCOPE) also protected tomato from *Pseudomonas syringae* infection (Figure 7E). The observation that CCOPEs can trigger IR in both a monocot and a dicot (Figure 1), indicates that the screening platform established by De Kesel et al. (2020) is not limited to the detection of IR stimuli for rice only. Nonetheless, rigorous *in planta* validation via plant infection experiments remains elementary to confirm the *in vitro* results obtained with this platform. Whereas other studies investigating cucurbit extracts for the control of nematodes uniquely focused on nematicidal activities (Elbadri et al., 2008; Gad et al., 2018; Alam and El-Nuby, 2019), we demonstrated that mCOPE combines nematicidal effects (Figure 3) and IR stimulation (Table 1 and Figure 4). Concerning the molecular mode-of-action of the triggered rice IR phenotype, we illustrated that mCOPE treatment leads to a systemic accumulation of the phytohormone ethylene (ET) (Figure 4E). This is in correspondence with the work of Nahar et al. (2011), who demonstrated that ET is essential for rice defense to *Mg*.

Although ROS are essential in plant defense signaling (Mittler, 2017), excessive levels of ROS can lead to severe cellular damage (Chapman et al., 2019). This makes ROS production and subsequent detoxification equally important for successful plant immunity (Chapman et al., 2019). Correspondingly, mRNA sequencing illustrated that ROS catabolism was systemically induced (Table 1), an observation that was validated by independent biochemical studies (Figure 4F). Similarly, rice root lignification upon foliar mCOPE treatment was indicated by the transcriptomic study (Table 1) and could be confirmed upon lignin quantification (Figure 4G). Moreover, the disturbed ROS homeostasis and induction of the phenylpropanoid pathway (Table 1) are indicative for root lignification (Passardi et al., 2004; Zhao, 2016; Waszczak et al., 2018). Nevertheless, the outcomes of the two latter biochemical assays require careful interpretation. Our data revealed a drop in ROS 1 day post mCOPE treatment (1 dpt), while no preceding accumulation was detected (Figure 4F). Arguably, the earliest moment of biochemical investigation (i.e., 6 h post treatment) may have already been tardy. However, as ROS such as H_2_O_2_ serve as input reagents for cell wall lignification (Passardi et al., 2004; Waszczak et al., 2018), the simultaneous induction of ROS catabolism and lignification may explain the observed ROS decrease at 1 dpt (Figure 4F). In accordance with this notion, Xue et al. (2015) demonstrated that accumulation/depletion of ferulic acid (i.e., an intermediate metabolite in lignin biosynthesis) led to lower/higher ROS levels, respectively. This illustrates the possibility of affected lignin biosynthesis rates disturbing ROS homeostasis or *vice versa*. Still, the transient peak in lignification as illustrated in Figure 4G is remarkable, as it is widely accepted that plants do not possess any lignin-degrading capacity (Barros et al., 2015). Nonetheless, similar findings concerning transient lignification have been reported by Ali et al. (2018) in the context of chitosan-IR establishment in dragon fruit (*Hylocereus polyrhizus*) plants. Alternatively, lignin-like compounds may have interfered with the deployed lignin quantification assay. Indeed, being degradation products of xylan, furfurals can be formed and detected by this assay (Hatfield et al., 1999; Moreira-Vilar et al., 2014). As hemicellulose, xylan contributes to cell wall recalcitrance, and thus to plant immunity (Rennie and Scheller, 2014). Interestingly, acetylation of xylan is a reversible cell wall state that affects plant immunity (Bacete et al., 2017) and influences the deconstruction rates of xylan into furfurals (Johnson et al., 2017). Specific quantification of xylan acetylation levels, assessment of lignification via alternative assays and/or a more profound analysis on ROS homeostasis may reveal the exact contribution of these elements to mCOPE-IR.

While a more profound characterization is needed, Figure 7 seems to indicate that mCOPE might have a predominant activity toward biotrophs. Similar results have been described by Walters et al. (2011). Aiming to protect barley (*Hordeum vulgare*) against *Blumeria graminis*, *Rhynchosporium secalis*, and *Ramularia collo-cygni* (being biotrophic, hemibiotrophic, and necrotrophic, respectively), the authors combined the IR stimuli benzothiadiazole (BTH), β-aminobutyric acid (BABA) and *cis*-jasmone. Only the former two pathogens were successfully controlled in a 2-year field experiment, while the abundance of the necrotroph under study was significantly increased (Walters et al., 2011). Moreover, Thaler et al. (1999) concluded that reduced susceptibility of tomato to *P. syringae* upon BTH treatment was associated with increased rates of herbivory damage caused by larvae of the beet armyworm (*Spodoptera exigua*). In our study, however, disease symptoms were not significantly increased for any of the evaluated pathosystems upon mCOPE-IR establishment (Figure 7).

Although we have provided multiple lines of evidence for mCOPE possessing both IR-inducing and nematicidal properties, its active ingredient(s) remain(s) unknown. As this is the first profound study on mCOPE, hypotheses concerning this/these ingredient(s) remain speculative. Nevertheless, the triterpenic cucurbitacins and/or the non-proteinaceous amino acid cucurbitin may be accountable for the nematicidal properties of mCOPE. While these respective components are predominantly and uniquely found in Cucurbitaceae plants and fruits (Miró, 1995; Chen et al., 2005; Wang et al., 2014), both have been suggested to be the responsible antihelmintic agent in various cucurbit extracts (Marie-Magdeleine et al., 2009; Dube and Mashela, 2016; Grzybek et al., 2016; Mashela and Shokoohi, 2021). With respect to the IR-triggering moiety, the range of tentative candidates is wide. Indeed, damage-associated molecular patterns (DAMPs) (Burketova et al., 2015), cell wall polysaccharides (Kidgell et al., 2019), mono- and disaccharides (Birch et al., 1993; Conrath, 2009; Kano et al., 2011; Trouvelot et al., 2014), organic metabolites (Doubrava et al., 1988) and inorganic salts (Inoue et al., 1994) have all been suggested and/or shown to be the active ingredient in plant-derived IR-triggering extracts. To identify mCOPE’s active ingredient(s), untargeted metabolome studies can be a promising and unbiased tool. This identification would allow minimal batch-to-batch variation and facilitate further translational research (Povero et al., 2016). Anyhow, with eco-friendly and/or waste stream-derived pest control agents being of high interest in nowadays agriculture (Xu and Geelen, 2018; Oka, 2020), the hereby presented data form a promising basis for future research and applications.

## Materials and Methods

### CCOPEs Preparation

Cucurbitaceae COld Peeling Extracts (CCOPEs) were made by blending 100 g of fresh fruit peeling in 200 mL sodium phosphate buffer (0.1 M; pH = 6.5). Only for the *in vitro* studies on rice cell suspension cultures (RCSCs), a mCOPE concentration of 200 g per 200 mL was used (see section “*In vitro* Evaluation of IR Activation”). Peelings were blended until a homogeneous mixture was obtained, which was then filtered using Miracloth filtration material. CCOPE preparation and eventual short-term storage was carried out at 4°C. For the *in vitro* analysis (see section “*In vitro* Evaluation of IR Activation”) and the nematicidal assays (see section “Nematicidal Assays”), extracts were filter-sterilized to avoid contamination.

### Plant Treatments

To establish IR, plants were foliarly treated with a watery solution of the IR stimulus under study. This was done 1 day before infection/inoculation. Only for the fodder beet-*Rhizoctonia solani* pathosystem, this procedure was not followed. Here, mCOPE treatment took place on the same day as fodder beet transplanting. All solutions were supplied with 0.02% (v/v) Tween20 as surfactant and sprayed via a fine mist until run-off. Via multiple application rounds with minimal time intervals, a total of 6.25 mL IR stimulus was applied per plant. Only for the infection assays conducted on strawberry plants, 15 mL CCOPE was used per plant. Control plants were treated with an equal volume of the buffer used for CCOPE preparation, also containing 0.02% (v/v) Tween20. When comparing the effectivity of mCOPE-IR in rice and tomato with commercial IR stimuli and/or nematicides, FytoSave^®^, and Actigard^®^ and Nemguard^®^ were dissolved in the buffer used for CCOPE preparation and contained 0.02% (v/v) Tween20. Fytosave^®^ was used at the recommended dose of 0.5% (v/v), which corresponds to 12.5 g/L COS-OGA. To study the effects of Actigard^®^, solutions with a concentration of 250 μM benzothiadiazole (BTH) were used. Unlike all other treatments, the nematicide Nemguard^®^ was administered as root drench. This was done 1 day before inoculation using a solution of 0.133 g/L and in accordance with the manufacturer’s instructions.

### *In vitro* Evaluation of Induced Resistance Activation

RCSCs were established and used for IR stimulus detection as described by De Kesel et al. (2020). First, rice seeds were sterilized subsequently for 5 and 30 min with 75% ethanol and a 5% HAZ TABS solution, respectively. After rinsing with sterile water, seeds were cultivated for 30 days on callus inducing medium (CIM) with pH = 5.8. Then, the obtained callus pieces were proliferated in liquid amino acid (AA) medium (pH = 5.8) using disposable CELLSTAR cell culture flasks. RCSCs were grown in the dark at 25°C whilst being shaken at 110 rpm. Every 5–7 days, cells were subcultivated in fresh AA medium. The composition of CIM and AA medium is listed in Supplementary Tables 3, 4, respectively.

At least 10 days after RCSC establishment and approximately 4 days after the most recent subcultivation, cells from multiple RCSCs were separated from the callus clumps using a 70 μm falcon cell strainer to be pooled. Per biological replicate, 37.5 mL cells were transferred to new cell culture flasks and 12.5 mL CCOPE was added hereto. The buffer used for CCOPE preparation was utilized to mock-treat the control RCSCs. After 4 h, cells were transferred to 50 mL tubes, centrifuged for 5 min at 4,000 × *g* and after removal of the supernatant snap-frozen in liquid nitrogen.

RNA extraction was done using TRI Reagent in combination with Direct-zol RNA purification columns. DNase-treatment was executed with DNase I and cDNA synthesis was done using the Maxima First Strand cDNA Synthesis Kit. RT-qPCR was performed with three technical replicates on two biologically independent replicates. A CFX Connect Real-Time PCR Detection System was used to execute the RT-qPCR cycles. Following PCR protocol was utilized: 10 min at 95°C, followed by 40 cycles of 25 s at 95°C, 25 s at 58°C, and 20 s at 72°C. Finally, a melting curve analysis was performed. Two reference genes were used to normalize for differences in cDNA input across all samples. All primer pairs are listed in Supplementary Table 5. Finally, statistical analysis was done using Rest2009 (Pfaffl, 2001).

### Plant Infection Experiments

#### Rice – *Meloidogyne graminicola*

*Oryza sativa* ssp. *japonica* cv. Nipponbare seeds were germinated for 4 days at 30°C on wet paper cloths. Then, seedlings were transferred into PVC tubes containing a mixture of quartz sand and a water absorbing polymer to be grown at 28°C under a 16 h/8 h light/dark regime as described by Sannier et al. (1999). Fifteen-days-old rice plants were inoculated with 250 *M. graminicola* (*Mg*) second-stage juveniles (J2s). Infection levels and growth parameters were evaluated 2 weeks post inoculation. To visualize galls and nematodes, root systems were boiled for 3 min in a 12.5% raspberry red solution. Afterward, roots were washed with tap water to be further destained in acid glycerol. Finally, galls and nematodes were counted per root system. At least two independent repetitions were conducted per rice-*Mg* infection experiment, with each experiment including minimally eight individual plants per treatment. All datasets resulting from rice-*Mg* infection assays were normalized in order to obtain similar data for the untreated control plants. This led to an average number of galls, number of females, root length (in cm) and shoot length (in cm) of 15, 40, 15, and 35 for the control plants, respectively. Resulting datasets were then combined and outliers were identified via the IQR-method to be removed (Barbato et al., 2011). Statistical differences were identified using a two-sided heteroscedastic *t*-test (*p* < 0.05).

To study long-term effects on growth and development of biweekly mCOPE treatment, plants were grown under greenhouse conditions as described by De Kesel et al. (2020). These plants were biweekly treated with water or mCOPE as described above. Long-term growth and yield performances were evaluated via two independent repetitions, consisting of twelve and nine plants, respectively. The data resulting from the repetitions were combined and normalized so that the control plants were on average 70 cm long, had, respectively, eight and three tillers and panicles, and resulted in a seed yield of 2 g per plant. Resulting datasets were then combined and outliers were identified via the IQR-method to be removed (Barbato et al., 2011). Statistical differences were identified using a two-sided heteroscedastic *t*-test (*p* < 0.05).

#### Tomato – *Meloidogyne incognita*

Seeds of *Solanum lycopersicum* var. *MoneyMaker* were germinated for 1 week at 24°C in moist potting soil. Seedlings were then transferred to a mixture of sieved potting soil and quartz sand in a 1:3 volume ratio to be grown at 24°C under a 16 h/8 h light/dark regime. Fifteen-days-old tomato plants were inoculated with 250 *M. incognita* (*Mi*) J2s. The infection level of the plants was evaluated 28 days post inoculation. To visualize galls and nematodes, root systems were stained similarly as for rice (see section “Rice – *M. graminicola*”). To evaluate the infection level, the number of galls was counted per root system. At least two independent repetitions were conducted per tomato-*Mi* infection experiment, with each experiment consisting of minimally eight individual plants per treatment. All datasets resulting from tomato-*Mi* infection assays were normalized in order to obtain similar data for the untreated control plants. This led to an average number of galls, root length (in cm), shoot length (in cm), and shoot dry weight (in mg) of 45, 20, 30, and 10 for the control plants, respectively. Resulting datasets were then combined and outliers were identified via the IQR-method to be removed (Barbato et al., 2011). Statistical differences were identified using a two-sided heteroscedastic *t*-test (*p* < 0.05).

#### Rice – *Pratylenchus zeae*

Rice plants were grown as described above. Root systems of 15-days-old plants were inoculated with 250 *Pratylenchus zeae* J2s. Infection levels were evaluated 25 days post inoculation. To visualize necrotic spots, root systems were boiled for 3 min in an acid fuchsin solution. Afterward, roots were washed with running tap water to be destained further in acid glycerol. To evaluate the infection level, the number of necrotic spots was counted per root system. One independent replicate study was conducted, with each treatment consisting out of eight individual plants. Outliers were identified via the IQR-method (Barbato et al., 2011) to be removed. Statistical differences were identified using a two-sided heteroscedastic *t*-test (*p* < 0.05).

#### Sugar Beet – *Heterodera schachtii*

Sugar beet (*Beta vulgaris* cv. Amarok) plants were grown to be inoculated with 300 *Heterodera schachtii* J2s at the age of 3 weeks as described by Singh et al. (2020a). Infection levels were evaluated 6 weeks post inoculation. Cysts were obtained by washing the infested soil, by letting them float in the washing water and subsequent handpicking with a small paintbrush. Consequently, the collected cysts were categorized either as empty (i.e., no eggs) or full (i.e., with eggs). One independent replicate study was conducted, with each treatment consisting out of eight individual plants. Outliers were identified via the IQR-method (Barbato et al., 2011) to be removed. Statistical differences were identified using a two-sided heteroscedastic *t*-test (*p* < 0.05).

#### Strawberry – *Botrytis cinereae*

Strawberry (*Fragaria* × *ananassa* cv. Elsanta) plants were grown in a greenhouse to be inoculated with *Botrytis cinerea* mycelium and conidiophores at the age of 12 weeks old as described by De Tender et al. (2016). Hereto, *B. cinerea* isolate 895 was cultured on potato dextrose agar at 20°C for 10 days under UV light. Each plant was infected by applying per leaf three 4 mm agar discs taken from the fungal plates. This was done for three leaves per plant. Afterward, plants were sprayed with water and covered with a plastic sheet to maintain high humidity. Disease state was rated at 5, 8, and 11 days post inoculation (5, 8, and 11 dpi, respectively). Hereto, a scoring system with a 0–4 scale based on infection area was used (0 = 0% of leaf area infected; 1 = 0–25% of leaf area infected; 2 = 26–50% of leaf area infected; 3 = 51–75% of leaf area infected; and 4 = 76–100% of leaf area infected). One independent replicate study was conducted, with each treatment consisting out of eight individual plants. Outliers were identified via the IQR-method [100] to be removed. Statistical differences were identified using a two-sided heteroscedastic *t*-test (*p* < 0.05).

#### Fodder Beet – *Rhizoctonia solani*

Fodder beet (*B. vulgaris* cv. Brunium) plants were grown until the age of 3 weeks. Then, plants were transferred to pots containing a 50/50 mixture of sand and light sandy loam. Per pot, sand/sandy loam had been mixed with 4 g of cereal grains to be inoculated with *Rhizoctonia solani* (anastomosis group 2-2 IIIB, isolates RS18.005, RS64 and RS85; obtained from the Experimental Farm in Bottelare, Belgium). The latter was done at the same day as fodder beet germination. Disease state was rated at nineteen, 26 and 47 days post inoculation (19, 26, and 47 dpi, respectively), using a scoring system with a 0–5 scale based on infection area (0 = no damage; 1 = minimal damage; 2 = moderate; 3 = severe damage; 4 = nearly dying plants and 5 = dead plants). One independent replicate study was conducted, with each treatment consisting out of eight individual plants. Outliers were identified via the IQR-method (Barbato et al., 2011) to be removed. Statistical differences were identified using a two-sided heteroscedastic *t*-test (*p* < 0.05).

#### Tomato – *Pseudomonas syringae*

Seeds of *Solanum lycopersicum* var. MoneyMaker were germinated for 1 week at 24°C in moist potting soil. Seedlings were transferred to potting soil only and grown at 25°C. Above-ground parts of 8-days-old seedlings were dipped in a *Pseudomonas syringae* solution having an OD of 0.25, containing 10 mM MgSO_4_ and 0.05 v/v% Silwet-77. Plants were placed back on 100% humidity for 24 h. Lesions were quantified 4 days post inoculation. Per individual repetition, each treatment consisted out of eight individual plants. At least two completely independent repetitions were conducted per tomato-*P. syringae* infection experiment. Each experiment consisted of minimally eight plants per treatment. For the sake of consistency throughout this work, all datasets resulting from tomato-*P. syringae* infection assays were normalized in order to obtain similar data for the untreated control plants. This led to an average number of lesions of 3, 3, and 0.5 on the first, second and third leaf, respectively, for the control plants. Resulting datasets were then combined and outliers were identified via the IQR-method to be removed (Barbato et al., 2011). Statistical differences were identified using a two-sided heteroscedastic *t*-test (*p* < 0.05).

### Nematicidal Assays

Nematicidal effects were evaluated by incubating approximately 100 *Mg* or *Mi* J2s in 1 mL of the test solution. As negative control, the buffer used for CCOPE preparation was utilized. Vertimec, a commercial formulation of the strongly nematicidal compound abamectin (Li et al., 2018), was used as positive control in a concentration of 0.2% (v/v). Per time point, at least three biological replicates were included. Nematode mortality was evaluated after six and 24 h of incubation. Nematodes were considered dead when they were not moving upon contact with a small picking needle. After identification and removal of outliers using the IQR-method (Barbato et al., 2011), statistical differences were determined via a heteroscedastic two-sided *t*-test (*p* < 0.05).

### mRNA Sequencing

Systemic transcriptomic alterations were studied via mRNA sequencing in roots of rice plants whose shoots had been treated 1 or 4 days earlier (1 and 4 dpt, respectively). Upon treatment, plants were 14 days old. Same-aged plants treated with the buffer used for CCOPE preparation were used as controls. For each condition, three biologically independent replicates were used, each consisting of at least four pooled root systems. Ground root material was used as input for RNA extraction. This was done using the RNeasy Plant Mini Kit according to the manufacturer’s protocol, with three additional sonication steps of 10 s each after addition of the RLT buffer.

The QuantSeq 3′ mRNA-Seq Library Prep Kit was used for RNA seq library preparation. Quality of the libraries was confirmed using an Agilent Bioanalyzer 2100 and used for sequencing on a NextSeq 500 Illumina sequencing platform. The samples were multiplexed to minimize lane effects. Via single end sequencing, reads of 76 nucleotides in length were generated. Unprocessed sequencing data can be retrieved at the repositories of NCBI (Geer et al., 2010) as BioProject PRJNA767540. Reads were trimmed with Trimmomatic (version 0.36) using following settings: ILLUMINACLIP:TruSeq3-SE.fa:3:30:10, SLIDINGWINDOW:5:20, MINLEN:20 (Bolger et al., 2014) and mapped against the *O. sativa* ssp. *japonica* reference genome (build MSU7.0) using STAR (version 2.5.2a) (Dobin et al., 2013). Only uniquely mapped reads were kept for further analysis. BAM files of multiplexed samples were merged using samtools (version 1.3). Count tables were generated by the “Summarize Overlaps” function in the Genomic Alignments R package (version 1.16.0) (Lawrence et al., 2013). Differential expression analysis was performed using the DESeq2 package (version 1.20) (Love et al., 2014). Relative gene expressions with an adjusted *p*-value < 0.05 were considered as differentially expressed.

Gene ontology (GO)-enrichment analyses were done using the g:Profiler tool on biit.cs.ut.ee/gprofiler/gost (Reimand et al., 2016). As such, GO annotations significantly overrepresented among the significantly differentially expressed genes could be identified (*p* < 0.05). MapMan analyses were executed to assess general pathway inductions (Thimm et al., 2004) via Wilcoxon signed rank tests using Benjamini and Hochberg corrected *p*-values (*p* < 0.05).

### Phytohormone Measurements

To quantify ethylene, foliar treatment with mCOPE was executed in order to collect roots 6 h post treatment (6 hpt), as well as at 1 and 4 dpt. Same-aged plants treated with the buffer used for CCOPE preparation were used as controls. Per biological replicate, four root systems were pooled. Per condition, eight replicates were analyzed. Roots were cut into small pieces and placed in glass vials, which were subsequently sealed to allow ET accumulation in the headspace. After 4 h of incubation at room temperature, the headspace was analyzed by gas chromatography with a flame ionization detector. Sampling was executed using a gastight syringe.

Abscisic acid (ABA), indole-3-acetic acid (IAA), jasmonic acid (JA) and salicylic acid (SA) levels were determined according to the protocol described by Haeck et al. (2018). After sampling, roots were ground in liquid nitrogen. This was followed by a cold solvent extraction, filtration and clean up. Ultimately, phytohormone levels were measured using an ultra-high-performance liquid chromatograph in combination with a tandem high resolution mass spectrometer. Five biological replicates were assayed, each consisting of a pool of at least four root systems.

After identification and removal of outliers using the IQR-method (Barbato et al., 2011), statistical differences were determined via a heteroscedastic two-sided *t*-test (*p* < 0.05).

### Quantification of Reactive Oxygen Species

Root ROS content were analyzed at 6 hpt, 1 dpt, and 4 dpt using the xylenol orange assay. Same-aged plants treated with the buffer used for CCOPE preparation were used as controls. Root material was ground in liquid nitrogen and 70–100 mg sample was transferred to an Eppendorf tube. Per mg biological matter, 10 μL of a 5% (m/v) trichloroacetic acid (TCA) solution was added. Next, samples were centrifuged for 10 min at 20,000 × *g* and at 4°C. Wells of a UV-transparent 96 well plate were filled with 100 μL buffer. This was a mixture of 100 volumes 100 mM sorbitol, 125 μM xylenol orange and 1% ethanol, and 1 volume 25 mM ferrous ammonium sulfate and 2.5 M sulfuric acid. Wells that were used as blanks contained a buffer without xylenol orange. Correspondingly, each sample was analyzed via both of the buffers. Hereto, 10 μL TCA extract was added to the wells. Finally, the plate was incubated in the dark for 30 min at room temperature and absorbance was measured at 560 nm using a Tecan Infinite F200 Pro machine. For the actual ROS measurement, two technical replicates were used, while for the blanks only one. Two replicate studies were conducted for this experiment. Per condition, eight biologically independent replicates were used. Each of them consisted out of three pooled root systems. After identification and removal of outliers using the IQR-method (Barbato et al., 2011), statistical differences were determined via a heteroscedastic two-sided *t*-test (*p* < 0.05).

### Lignin Quantification

Lignin levels in rice roots were quantified at 1 and 4 dpt using an acetyl bromide assay. Same-aged plants treated with the buffer used for CCOPE preparation were used as controls. Samples were first ground in liquid nitrogen. Then, they were incubated in 1 mL of a series of solvents at a specific temperature, followed by centrifugation for 3 min at 20,000 × *g* and removal of the supernatant. The solvent - temperature combinations were, in following order, water −98°C, 96% ethanol −76°C, chloroform −59°C, and acetone −54°C. Next, the samples were left to dry, weighed to assure a mass between 2 and 7 mg and dissolved in 40 μL glacial acetic acid per mg dried tissue. The acetic acid contained 25% acetyl bromide and samples were incubated for 2 h at 50°C. Next, 1 mL glacial acetic acid was added to each sample, followed by centrifugation at 20,000 × *g* for 10 min. 300 μL of the resulting samples was mixed with 300 μL 2 M sodium hydroxide and 300 μL 0.5 M hydroxylamine-hydrochloride. Finally, the absorption of this mixture was measured at 280 nm using a Tecan Infinite F200 Pro machine, using three technical replicates per sample. Per treatment, eight biological replicates were used, each consisting out of four pooled root systems. After identification and removal of outliers using the IQR-method (Barbato et al., 2011), statistical differences were determined via a heteroscedastic two-sided *t*-test (*p* < 0.05).

## Data Availability Statement

The original contributions presented in the study are publicly available. This data can be found here: National Center for Biotechnology Information (NCBI) BioProject database under accession number PRJNA767540.

## Author Contributions

TK, JDK, and ED: conceptualization. JDK: formal analysis, data curation, writing—original draft preparation, and visualization. JDK, ED, RN, RS, KD, KDK, RA, JM, and EW: investigation. TK, JDK, ED, RS, KD, JM, EW, and JD: writing—review and editing. TK, GH, and JD: supervision. TK: funding acquisition. All authors contributed to the article and approved the submitted version.

## Conflict of Interest

The here-reported Cucurbitaceae COld Peeling Extracts (CCOPEs) have been patented by TK, JDK, and ED (Kyndt et al., 2021). The remaining authors declare that the research was conducted in the absence of any commercial or financial relationships that could be construed as a potential conflict of interest.

## Publisher’s Note

All claims expressed in this article are solely those of the authors and do not necessarily represent those of their affiliated organizations, or those of the publisher, the editors and the reviewers. Any product that may be evaluated in this article, or claim that may be made by its manufacturer, is not guaranteed or endorsed by the publisher.
