# Supplementary material for: Cucurbitaceae COld Peeling Extracts (CCOPEs) Protect Plants From Root-Knot Nematode Infections Through Induced Resistance and Nematicidal Effects
Source: Front Plant Sci. 2022 Jan 26;12:785699. doi: 10.3389/fpls.2021.785699 (PMC8826469; doi:10.3389/fpls.2021.785699)
Supplement: Supplementary file 6 [file Data_Sheet_1.DOCX]

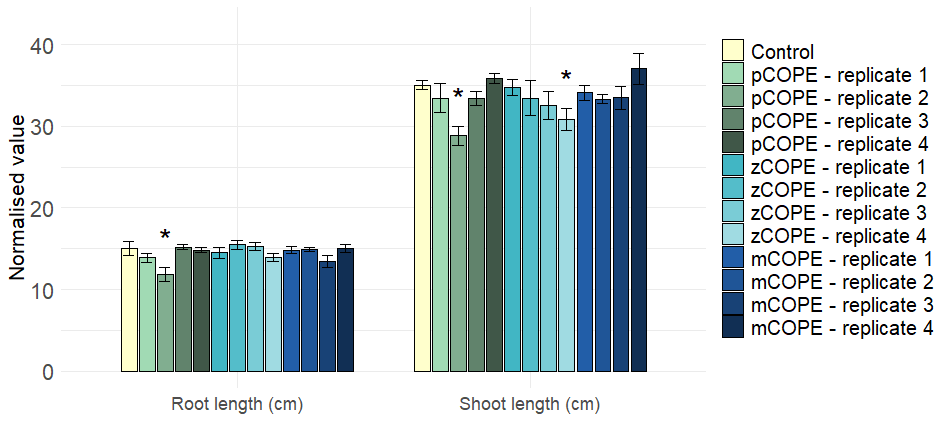


**Supplementary Figure 1.** *Cucurbitaceae* COld Peeling Extract (CCOPE) derived from pumpkin (*Cucurbita moschata* cv. Musquee de Provence; pCOPE) or zucchini (*Cucurbita pepo* var. *cylindrica*; zCOPE) sporadically leads to impaired growth performances of rice plants upon foliar treatment. CCOPE derived from melon (*Cucumis melo* var. *cantalupensis*; mCOPE), on the other hand, was never found to hamper plant growth. Normalized root and shoot lengths of one-month-old rice plants are illustrated, as assessed upon termination of *Meloidogyne graminicola* infection experiments. Each bar represent the outcome of one biologically independent replicate study, resulting from an analysis on minimally eight plants. For the sake of clarity, data of only one representative control group (treated with the buffer used for CCOPE preperation) are illustrated (Control). Even so, all biologically independent replicate studies contained an individual group of control plants and statistical comparisons were consistently done with respect to the within-experiment controls. Error bars represent the standard error of the mean. Asterisks indicate significant differences, determined via a two-sided heteroscedastic t-test (p < 0.05).
