# Supplementary material for: Cucurbitaceae COld Peeling Extracts (CCOPEs) Protect Plants From Root-Knot Nematode Infections Through Induced Resistance and Nematicidal Effects
Source: Front Plant Sci. 2022 Jan 26;12:785699. doi: 10.3389/fpls.2021.785699 (PMC8826469; doi:10.3389/fpls.2021.785699)
Supplement: Supplementary file 7 [file Data_Sheet_2.DOCX]

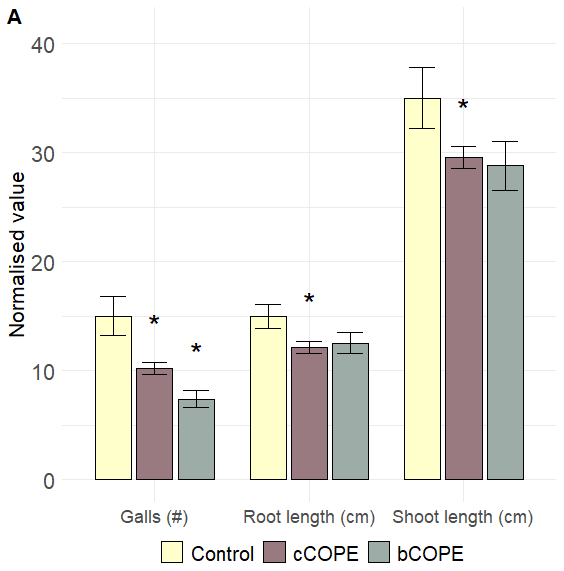

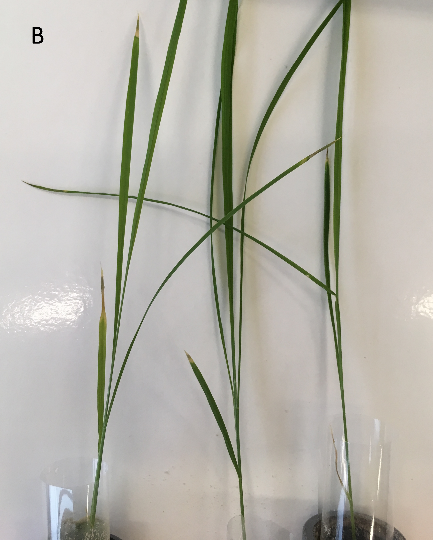

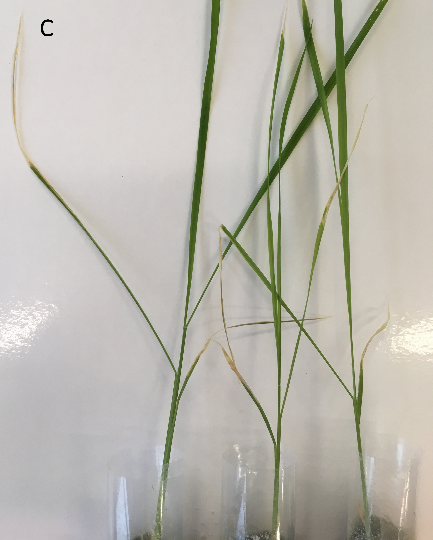


**Supplementary Figure 2.** *Cucurbitaceae* COld Peeling Extract (CCOPEs) derived from cucumber (*Cucumis sativus*; cCOPE) or butternut pumpkin (*Cucurbita moschata* cv. Butternut; bCOPE) trigger an IR phenotype that lowers the susceptibility of rice to *Meloidogyne graminicola*, but also lead to impaired growth performances and/or phytotoxicity. **(A)** Infection and growth parameters, as assessed for rice plants fourteen days after inoculation with 250 *Mg* second stage juveniles. One day before inoculation, shoots of fourteen-days-old plants were treated with the buffer used for CCOPE preparation (Control), cCOPE or bCOPE. Error bars represent the standard error of the mean. Asterisks indicate significant differences upon comparison of CCOPE-treated plants with mock-treated control plants. Statistical differences were determined via a two-sided heteroscedastic t-test (p < 0.05). **(B-C)** Whilst Supplementary Figure 2A does not indicate a significant difference concerning root or shoot length for bCOPE-treated plants upon comparison with control plants, Supplementary Figure 2B (displaying control plants) and Supplementary Figure 2C (displaying bCOPE-treated plants) clearly demonstrate the phytotoxicity induced by this extract. Pictures were taken fourteen days after inoculation with 250 *Mg* second stage juveniles. One day before inoculation, shoots of fourteen-days-old plants were treated with **(B)** the buffer used for CCOPE preparation or **(C)** bCOPE.
